# Timely estimation of National Admission, readmission, and observation-stay rates in medicare patients with acute myocardial infarction, heart failure, or pneumonia using near real-time claims data

**DOI:** 10.1186/s12913-020-05611-w

**Published:** 2020-08-10

**Authors:** Shu-Xia Li, Yongfei Wang, Sonam D. Lama, Jennifer Schwartz, Jeph Herrin, Hao Mei, Zhenqiu Lin, Susannah M. Bernheim, Steven Spivack, Harlan M. Krumholz, Lisa G. Suter

**Affiliations:** 1 Section of Cardiovascular Medicine, Department of Internal Medicine, Yale School of Medicine, New Haven, CT USA; 2 Center for Outcomes Research and Evaluation, Yale-New Haven Health System, New Haven, CT USA; 3 National Opinion Research Center University of Chicago, Washington, District of Columbia USA; 4 UC San Diego Health, San Diego, CA USA; 5 Department of Health Policy and Management, Gillings School of Public Health, Univeristy of North Carolina, Chapel Hill, NC USA; 6 Department of Health Policy and Management, Yale School of Public Health, New Haven, CT USA; 7 Section of Rheumatology, Department of Internal Medicine, Yale School of Medicine, New Haven, CT USA; 8 West Haven Veterans Administration Medical Center, West Haven, CT USA

**Keywords:** Real-time reporting, Prediction models, Medicare claims data, Readmission, Observation stay

## Abstract

**Background:**

To estimate, prior to finalization of claims, the national monthly numbers of admissions and rates of 30-day readmissions and post-discharge observation-stays for Medicare fee-for-service beneficiaries hospitalized with acute myocardial infarction (AMI), heart failure (HF), or pneumonia.

**Methods:**

The centers for Medicare & Medicaid Services (CMS) Integrated Data Repository, including the Medicare beneficiary enrollment database, was accessed in June 2015, February 2017, and February 2018. We evaluated patterns of delay in Medicare claims accrual, and used incomplete, non-final claims data to develop and validate models for real-time estimation of admissions, readmissions, and observation stays.

**Results:**

These real-time reporting models accurately estimate, within 2 months from admission, the monthly numbers of admissions, 30-day readmission and observation-stay rates for patients with AMI, HF, or pneumonia.

**Conclusions:**

This work will allow CMS to track the impact of policy decisions in real time and enable hospitals to better monitor their performance nationally.

## Background

Medicare provides health care coverage for over 60 million Americans [1]; over 95% of those are over the age of 65 [2]. The information available in Medicare claims, therefore, offers a detailed and comprehensive view of patterns of health and healthcare for older Americans. Claims data are frequently used by researchers and government to examine trends in disease, healthcare utilization, and quality [3, 4]. Theoretically, these claims could also provide critical early insight on changing disease patterns or allow for monitoring of short term changes in care patterns, including responses to policy changes. However, limited access to claims and delays in obtaining complete and accurate claims have limited the development of early warning systems [5]. For example, to track critical information from hospital claims, it can take up to a year for the accrual of all the final claims for a particular period [6] (i.e., for patients admitted in January, providers have until December to submit the final claim to CMS for payment). However, during this time of final claim accrual, as initial claims are submitted and processed, new information is accumulating that may be a viable source of prediction for concurrent and future claims and care patterns.

We therefore sought to examine the claims data that becomes available in real-time and to use early claims data to predict the findings of the complete data that would be finalized in later months. We sought to predict national rates of readmission and observation stay usage within a short-window following initial hospitalizations. We theorized that we could build models that would accurately predict, based on early submitted claims, what the final set of claims would show about utilization rates, much as is done in early voting returns predicting election results. Tracking national readmission rates and use of observation stays in real-time responds to direct needs of both the Centers for Medicare and Medicaid Services (CMS) and individual hospitals. Such a system will allow CMS to track the impact of policies; patterns that suggest improvements in care or worrisome trends for patients can be identified and responded to more rapidly. For hospitals that track their own readmission rates in response to pay-for-performance programs such as the Hospital Readmission Reduction Program (HRRP) [7], this approach can provide information about comparative national performance on a more rapid cycle. Currently, hospitals rely on CMS’s annual updates of measure results which reflect the data from a year or more earlier [6].

This article details the data sources, methodology, and results of newly developed real-time reporting models for estimating national numbers of admissions, 30-day unplanned readmission rates, and 30-day post-discharge observation-stay rates for patients with acute myocardial infarction (AMI), heart failure (HF), or pneumonia using final and non-final claims data. We aligned our methodology with that used by CMS for public reporting of 30-day unplanned readmissions after hospitalization for AMI, HF, or pneumonia. In this article, we examined the ability of predictive models to accurately forecast the findings of complete claims, an approach that could have wide-spread use if successful.

## Methods

### Overview

We aligned our methodology with that used by CMS for public reporting of 30-day unplanned readmissions after hospitalization for AMI, HF, and pneumonia to ensure our results were policy relevant. We focused on AMI, HF, and pneumonia because they were the first three conditions targeted by CMS’s HRRP. Using Medicare administrative claims data, we created training, test and validation datasets for development and testing of our predictive models. We used training and test datasets to ensure that the model has internal validity; and the validation dataset, which is independent from the prior two datasets, to ensure external validity and that there is no model overfit. We used an identical approach to model development across AMI, HF, and pneumonia cohorts. For simplicity, we present AMI in the main report. Parallel findings for HF and pneumonia are presented in the Additional file 1.

To develop our models, we first identified Medicare fee-for-service (FFS) patients admitted with a principal discharge diagnosis of one of the target conditions (AMI, HF, or pneumonia) and examined patterns of delay in claims accrual, including evaluating the impact of using final versus non-final action claims. A final action claim is determined to be the final representation of the claim submission, while non-final action claims are still subject to adjustment before being finalized. We used historical data to develop, test and internally validate six time-series models for each condition (AMI, HF, and pneumonia) based on autoregressive integrated moving average (ARIMA) methods to estimate values for the most recent six-month period for each outcome (number of admissions, 30-day readmission rates, and 30-day post-discharge observation-stay rates). ARIMA is a commonly used statistical analysis model that uses time series data to either better understand the data set or predict future trends [8]. Next, we conducted look-back validation of each time-series model by comparing the estimated rates obtained from the model with the final rates obtained from data downloaded from the Integrated Data Repository (IDR, see below) in later years. The approach described below for AMI was later applied to HF and pneumonia (see Additonal file 1).

### Data source (Fig. 1)

**Fig. 1:**
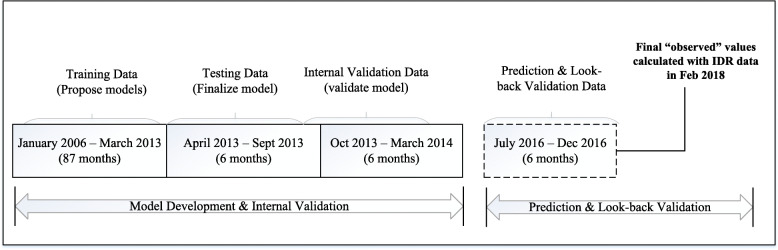
Datasets used for model development, prediction, and validation. Display of the IDR claims data sets used for RTR model development, validation, prediction and look-back validation

The CMS IDR is a data warehouse that contains Medicare parts A, B, C, D and durable medical equipment (DME) claims data since 2006 [9]. Claims are continuously uploaded into the IDR, allowing for immediate access to the most recently submitted claims, including both final and non-final action claims data. We accessed the IDR claims data via the Medicare Virtual Data Mart for the overall trend and estimation of the outcomes for the three conditions. For the AMI cohort, we initially developed and validated predictive models for the three outcomes using data downloaded from the IDR as of June 2015 (January 2006 – March 2014). We then used these models to estimate monthly admissions, readmission rates and observation-stay rates in later years (July 2016–December 2016) using data downloaded from the IDR in February 2017 (reflecting the two-month delay to capture 30-day events for admissions through December 31, 2016). Finally, we conducted look-back validation of the estimated rates for July 2016 through December 2016 using data downloaded from the IDR in February 2018 (Fig. 1). The IDR stores all versions of claims for the same admissions with processing time stamps and status codes. We utilized both final and non-final action claims for identifying cohorts and outcomes. When multiple versions of claims existed for the same admission, we used the latest version.

### Cohort (denominator) definitions

For each month, we identified Medicare FFS claims discharged with a principal diagnosis of AMI from short-term acute-care or critical access hospitals (Additonal file 1 Fig. A1). Disease condition cohorts were identified using International Classification of Diseases, Ninth Revision, Clinical Modification (ICD-9-CM) codes for claims filed before Oct 1, 2015 and ICD-10-CM codes thereafter. We excluded claims for patients age ≤ 65, transferred out of the hospital, discharged against medical advice, or that died in the hospital. Claims within 30 days of a prior qualified admission were excluded. These inclusion and exclusion criteria are consistent with the publicly reported readmission measures [10].

### Outcome (numerator) definition and timing

We calculated the monthly number of admissions. We also calculated observed 30-day unplanned readmissions and post-discharge observation stays as dichotomous (yes/no) outcomes. Both readmission and observation stay outcomes were summarized as monthly rates for analysis. In accordance with the publicly reported measures, we excluded planned readmissions which were defined using a vetted and validated algorithm from the readmission outcomes [11]. We grouped monthly admissions based on discharge dates. For example, the month of February 2014 counts all the admissions that have a discharge date in the month of February 2014, regardless of admission month.

Consistent with our prior work on observation stays and the definition used for surveillance assessment in the CMS *Medicare Hospital Quality Chartbook* (2014) [12], we identified observation-stay claims using the Healthcare Common Procedure Coding System (HCPCS) code G0378, found in the outpatient claim line data file. This definition aims to identify unscheduled and unplanned observation stays that are most likely to represent care that is similar to what patients receive during short inpatient admissions and hence, might reflect potential substitutions for inpatient readmissions [6].

Patients may experience multiple acute-care visits within the 30-day post-discharge period. Outcomes were defined hierarchically so that each hospitalization could be counted in only one of the three post-discharge care event categories (unplanned readmission, observation-stay without any associated readmission, or no post-discharge care event (Additonal file 1 Fig. A2). A patient with a post-discharge observation stay within 30 days after discharge was considered to have had a post-discharge observation stay only if he or she had not *also* experienced an unplanned readmission within 30 days after discharge.

Based on claims available from the IDR, we calculated the monthly 30-day readmission and observation-stay rates from January 2006 until the 2 months prior to the month data were available/downloaded from the IDR. We needed to wait at least 2 months after the discharge date of an index admission during a given month, since our measurement period is 30 days and estimating rates sooner would prevent patients admitted at the end of the month from having a complete 30-day follow-up for outcome ascertainment. For example, for patients with AMI discharged during July, eligible 30-day readmissions will occur throughout July and August, and the monthly readmission rate for July can only be calculated after August 30th; hence we waited until September 1st or later.

### Patterns of delay in claims accrual

Before developing our estimation models, we examined how rapidly claims accrued in the IDR, how rapidly they were finalized, and what changed between initial claims and final action claims. We found that the median time from discharge to claim submission is 17 days. We determined that for an index month, roughly 97% of claims are uploaded and finalized to the IDR within 7 months after the index month. Therefore, readmission rates calculated 8 months after the beginning of an index month will not be estimated and considered “predicted final”, because almost all claims for that index month have been submitted. For example, 97.0% of claims for Feb 1, 2013 have been finalized by October 1, 2013 (or 8 months after February 1, 2013; Additonal file 1 Table A1). Therefore, we created our models to estimate outcomes within the most recent six-month period. For instance, using data downloaded on February 2017, we considered the claims for any patients from June 2016 and prior as final, and sought to use these finalized months to estimate numbers and rates for months July 2016–December 2016. We also determined that claim revisions do not influence the principal discharge diagnosis or other variables used to define the measure cohorts or outcomes. Therefore, we decided to include all available claims (both final action and non-final action) for our monthly rates.

### Model development

To facilitate descriptions of our modeling approach, we used the following notations to represent our data and estimation goals for monthly numbers of admissions, 30-day readmission rates, and 30-day post-discharge observation-stay rates.

- *c:* number of months elapsed between January 2006 to the month data were downloaded, inclusive of download month. For example, when the data were downloaded from the IDR in February 2017, c = 134.
- *D*_*m*_*(t):* Monthly number of admissions at *t*^*th*^ month from January 2006, observed at *m*^*th*^ month after t; m = 2,^…^,8; *t = 1,*^*…*^*,c-2*. The model considers *D*_*8*_*(t)* as the final (true) number of admission for month *t*. For m = 2,^..,^7, *D*_*m*_*(t)* is based on non-finalized data, and must be estimated; ${\hat{D}}_m(t)$ denotes the estimated number of admissions.
- *R*_*m*_*(t):* 30-day readmission rate at *t*^*th*^ month from January 2006, observed at *m*^*th*^ month after t; m = 2,^…^,8; *t = 1,*^*…*^*,c-2*. The model considers *R*_*8*_*(t)* as the final (true) readmission rate for month *t*. For m = 2,^..,^7, *R*_*m*_*(t)* is based on incomplete data, and must be estimated; ${\hat{R}}_m(t)$ denotes the estimated rate.
- *O*_*m*_*(t):* 30-day post-discharge observation-stay rate at *t*^*th*^ month, counting from January 2006, observed at *m*^*th*^ month after t; m = 2,^…^,8; *t = 1,*^*…*^*,c-2*. For example, *O*_*8*_*(13)* is the rate of 30-day post-discharge observation stays for January 2007 (month 13 counting from January 2006) when observed 8 months later, in August 2007. The model assumes that the rate stabilizes at m = 8; that is, the model considers *O*_*8*_*(t)* as the final (true) rate for month *t*. For m = 2, ^…^,7, *O*_m_(*t*) is based on incomplete data, and must be estimated; ${\hat{O}}_m(t)$ denotes the estimated rate.
- *H*_*r*_*(t)/H*_*o*_*(t)/H*_*d*_*(t):* Indicates whether the calendar month readmission rates, post-discharge observation-stay rates, and number of admissions are historically above the annual monthly average (“high”). These were used as candidate covariates in estimation model development. To arrive at the annual monthly average, we first calculated the average of the outcomes in each of the 12 months using all finalized monthly data; then we calculated the average of those 12 monthly averages. For example, we calculated the average monthly readmission rate for January through December; the annual monthly average was calculated as the average of those 12 averages.

The goals were to estimate, for c = 8,..,134, the number of discharges for the six most recent months: *D*_*8*_*(c-7), D*_*8*_*(c-6), D*_*8*_*(c-5), D*_*8*_*(c-4), D*_*8*_*(c-3), D*_*8*_*(c-2);* six rates for readmission: *R*_*8*_*(c-7), R*_*8*_*(c-6), R*_*8*_*(c-5), R*_*8*_*(c-4), R*_*8*_*(c-3), R*_*8*_*(c-2);* and six rates for observation stays*: O*_*8*_*(c-7), O*_*8*_*(c-6), O*_*8*_*(c-5)*, *O*_*8*_*(c-4)*, *O*_*8*_*(c-3), O*_*8*_*(c-2)*.

We illustrated the overall approach, using readmission as an example in Additonal file 1 Fig. A3. Here, as of February 2017, we considered every readmission rate calculated for June 2016 or earlier as a final rate. We then built six separate models to predict readmission rates from July 2016 through December 2016 based on historical patterns of incomplete and final data (January 2006 through June 2016 data). To build and test the estimation models, we divided all claims data into three datasets. Specifically, the models for AMI, which were initially developed in 2015, used: 1) Training Dataset (January 2006 to March 2013; 87 months); 2) Test Dataset (April 2013 to September 2013; 6 months); and 3) Validation Dataset (October 2013 to March 2014; 6 months; See Fig. 1). The application of the datasets is described under Model Selection, below.

Our models are seasonal ARIMA-based and allow covariates and transformation of dependent variables. Table 1 summarizes our candidate models for the three different outcomes, including the dependent variable transformation function, information criteria for model selection, candidate covariates considered, and ARIMA parameter ranges.

**Table 1:** Candidate model sets for three different outcomes

	Number of admissions	30-day readmission rate	30-day observation-stay rate
**Dependent variables**	*D*_*8*_*(t)*	*R*_*8*_*(t)*	*O*_*8*_*(t)*
**Transformation function**	*Log* vs. *identity*	*Log* vs. *identity*	*Log* vs. *identity*
**Information criterion**	AIC or BIC		
**Candidate covariates**	*D*_*m*_*(t)* or *H*_*d*_*(t), m = 2,…,7*	*D*_*m*_*(t), R*_*m*_*(t),* or *H*_*r*_*(t), m = 2,…,7*	*D*_*m*_*(t), R*_*m*_*(t),O*_*m*_*(t),* or *H*_*m*_*(t), m = 2,…,7*
**ARIMA (*p,*d,q) x (P, D,Q)S**	p < =5, q < =5; P < =5,Q < =5 where S = 12 months		

Methodology summarizing the candidate models for the three different outcomes, including the dependent variable transformation function, information criteria for model selection, candidate covariates considered, and ARIMA parameter ranges. *AIC* Akaike information criteria, *ARIMA* Autoregressive integrated moving average, *BIC* Bayesian information criteria

### Model selection

To select final estimation models, we first used the Training Dataset (January 2006 to March 2013) to determine the seasonal ARIMA model parameters in combination with different sets of covariates based on Akaike or Bayesian information criteria using the R auto.arima(). Next, we used the Test Dataset (April 2013 to September 2013) to choose the covariates and dependent variation transformation function by examining several error terms, including mean error, root mean square error, mean absolute error, mean percentage error, mean absolute percentage error, and mean absolute scaled error. After considering these parameters and the tradeoffs between computational simplicity and robustness, we selected a final model for each prediction time point for each condition and outcome.

### Internal validation

To validate each of the final models, we used them to make monthly estimations for the period covered by the Validation Dataset (October 2013 to March 2014). We plotted observed and estimated values (with 95% confidence intervals [CIs]) to assess three indicators of model performance: 1) majority of observed values fall within the estimated CIs; 2) CIs form a trumpet shape in which the estimations for months with more data should be narrower than those for more recent months; and 3) all estimated point estimates are close to observed values.

### Model prediction

After defining the models, the overall timing of prediction is shown in Additonal file 1 Fig. A4. For each month that we needed a prediction, we use the chosen seasonal ARIMA parameter configuration, such as choice of P, D, Q, p, d, q, covariates, link function, and information criteria, to determine the updated coefficients for prediction (Table 1). We then calculate actual trends for monthly AMI admissions, readmissions, and observation-stay rates.

### Look-back validation

To ensure that the models still perform well in years following the initial development, we performed a retrospective look-back validation, comparing the results using data downloaded in February 2017 and again in February 2018 (which contains additional, updated and finalized claims to provide gold standard or reference rates for model validation, Fig. 1). First, we predicted the number of admissions, 30-day readmission rates, and post-discharge observation-stay rates from July 2016 through December 2016 using IDR data downloaded in early February 2017 (reflecting the two-month delay to capture 30-day events for admissions through December 31, 2016). Then we calculated those rates again using ‘gold standard’ data accessed from the IDR in in February 2018. We compared the estimated rates to see how well the models performed.

## Results

### Model development and testing

Based upon testing in the Training and Testing Datasets, we selected final models that demonstrated the least error with the greatest computational simplicity to estimate monthly national numbers of admissions, readmission rates, and observation-stay rates. The final model specifications for AMI, HF and pneumonia are presented in the Additonal file 1. We chose seasonal ARIMA configurations, either among the three different outcomes or within each outcome for different prediction timepoints. Generally, the more complete the data were the simpler the ARIMA form. For example, the model to predict the number of admissions only one month from having complete/finalized data, *D*_*8*_*(c-7)*, is a simple first-order auto-regressive (AR1) model with one covariate, *D*_*7*_*(t),* the historical values observed at the corresponding observation time. In contrast, the model for predicting, *D*_*8*_*(c-2),* which has more incomplete data, is a more complex ARIMA (2,0,0)(1,0,0) model with a seasonal term with non-zero mean and two covariates. Among the three outcomes, the models for 30-day observation-stay rates are the simplest, with five out of six models being simple linear regression models.

### Internal validation

**Fig. 2:**
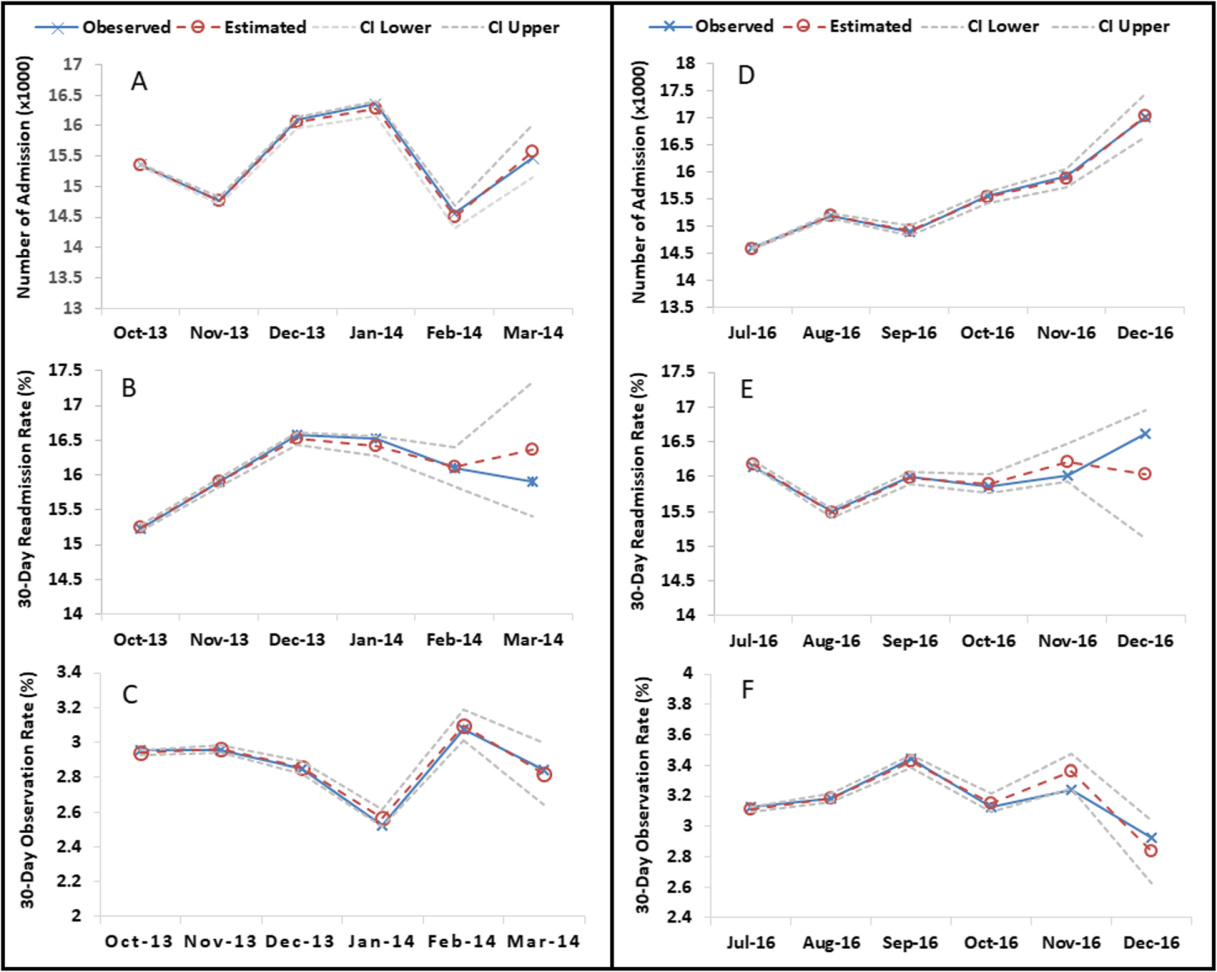
Internal and look-back validation for AMI outcomes. Panels A, B and C are for internal validation using validation data from October 2013 through March 2014 for number of admissions (**a**), 30-day readmission rates (**b**) and 30-day observation-stay rates respectively (**c**). Panels D, E and F are for look-back validation, where we compare the rates estimated for the months July 2016 through December 2016 using RTR models in February 2017 with the final rates later observed using data downloaded from the IDR in February 2018 for number of admissions (**d**), 30-day readmission rate (**e**) and 30-day observation-stay rate (**f**) respectively

When we developed the models, we verified the prediction models using the Validation Dataset, October 2013 through March 2014 (Fig. 2, Panels A-C). For all three outcomes – predicted number of admissions, readmission, and observation-stay rates. The values are very close to the observed (true/final) values and within the bounds of the 95% CIs of the predicted values. Also, the CIs resemble the shape of a trumpet, showing that the fewer the data, the greater the chance of having potential prediction errors.

### Look-back validation

In our look-back validation, all three outcomes predicted for July through December 2016 are very close to the observed (true/final) values and within the bounds of 95% CIs of the predicted values (Fig. 2, Panels D-F, with additional results in Additonal file 1). We observed very similar results for both look-back validation and internal validation. Thus, we concluded that the model continues to perform well over time. Look-back validation for the HF and pneumonia cohorts showed similar results (Additonal file 1).

### Monthly trend and model prediction

**Fig. 3:**
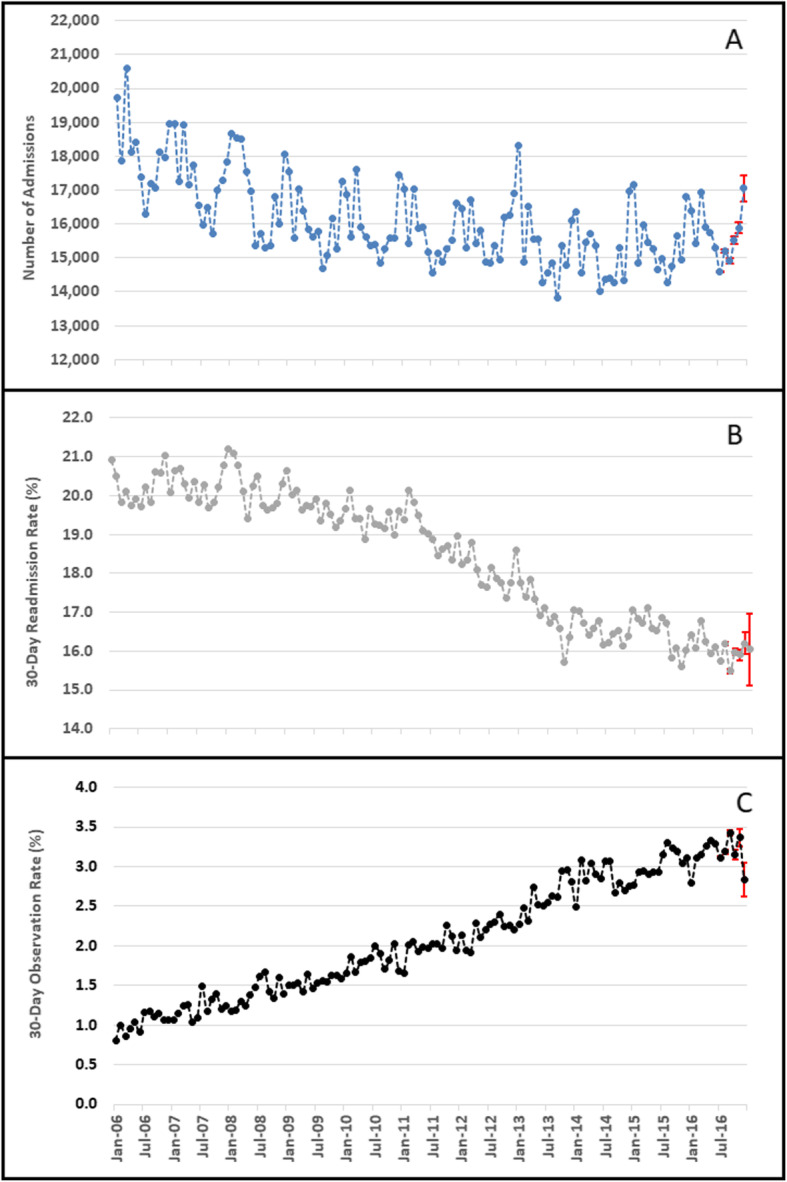
Trends for AMI monthly admissions (**a**), readmission (**b**) and observation-stay rates (**c**) from 2006 through 2016. Using data that was accessed from the IDR in February 2017, the figure shows the monthly trends for the numbers of admissions, readmission rates, and observation-stay rates for AMI from January 2006 through December 2016. The red whisks for the last six data points at each panel are for 95% estimation confidence intervals

Figure 3 shows the monthly trends for the numbers of admissions, readmission rates, and observation-stay rates from January 2006 through December 2016 using data accessed from the IDR in February 2017. The outcome rates from July 2016 to December 2016 were predicted using the models described in Additonal file 1 Table A2. The number of AMI admissions dropped slightly over time, with seasonal variations of having more admissions during winter months and fewer during summer months. The number of AMI admissions in December 2016 was predicted to be 17,050 (95% CI: 16653–17,446). The readmission rates decreased over time and began plateauing in late 2016. The December 2016 readmission rates were predicted to be 16.04% (95% CI: 15.12–16.96%). However, the observation-stay rates continued to increase through the end of 2016, fluctuating slightly each month and reached 2.83% (95% CI: 2.63–3.04%) in December 2016. For all cohorts, predicted readmissions showed the least precision, while admissions and observation stays more closely mirrored predicted numbers (Fig. 3).

## Discussion

We developed an approach for using early claims data to predict the findings of the complete data that would be finalized in later months. These models provide accurate, validated estimates of monthly numbers of admissions, 30-day readmission rates, and 30-day post-discharge observation-stay rates for patients discharged with AMI, HF, or pneumonia for as recently as 2 months after admission. Together, these models and their results provide a more complete picture of acute-care utilization within the 30-day post-discharge period for Medicare beneficiaries hospitalized for AMI, HF, or pneumonia. Since CMS’s publicly reported measures rely on claims data that are more than a year old, it impedes their ability to assess national outcomes in real time and to evaluate the possible impact of programs in a timely manner. Public deployment of these tools will allow CMS and hospitals to track and monitor national, unadjusted monthly readmission and observation-stay rates for these health conditions.

Other surveillance efforts that use estimation or predictive models have been reported [13], but none is specifically aligned with CMS’s publicly reported hospital outcome measures. Therefore, CMS and hospitals cannot adequately ascertain how those results affect hospital-performance measurement and payment programs. Providing CMS and hospitals with real-time national outcomes data support rapid evaluation of the effects of policy changes and enable individual hospitals to compare their internal readmission performance data against national rates.

Nevertheless, our work has some limitations. First, our approach requires a comprehensive, real-time data source such as the IDR and, therefore, could only be implemented and publicly reported by CMS. Second, we chose to select and implement models that minimized estimation error while still maintaining a low level of computational complexity; there may be models that could be developed to provide greater accuracy at the cost of additional computing burden. However, our models were proved to perform quite robustly over time. Further, much of the work required to implement these models and update the results on a monthly basis can be automated, minimizing the resources required to make these data public. The models may also need to be revised periodically for two reasons. First, CMS claims processes often change over time; the current models assume relatively stable claim accrual patterns. Drastic changes in either coding or clinical care, such as those due to the current coronavirus pandemic, may violate this assumption and invalidate the models. Second, the outcome and cohort definitions used for these tools may need be revised to maintain alignment with CMS’s publicly reported measures. Third, we use a unified approach for all three outcomes to streamline production. It is possible that greater specificity by outcome could increase precision further for, for example, the readmission models. Readmissions may be harder to predict because claim accrual patterns impact both numerator (outcome) and denominator (cohort). Further, readmission itself is a more discretionary outcome and may thus be harder to predict readmission rates than numbers of admissions or deaths.

## Conclusion

In summary, we created models that use real time CMS claims data to accurately estimate national observed numbers of admissions, 30-day readmission rates, and 30-day post-discharge observation-stay rates for patients discharged with AMI, HF, and pneumonia for as recently as 2 months. They represent the only such models that are fully harmonized with CMS publicly reported readmission measures and provide CMS and hospitals with powerful tools for real-time surveillance of national outcomes.

## Supplementary information

**Additional file 1: Figure A1**. Monthly cohort definition for AMI, HF or Pneumonia Readmissions and Observation Stays (Pg.2). **Figure A2.** Hierarchy for multiple post-discharge care events (Pg. 2). **Table A1.** Cumulative numbers and percentages of final action inpatient claims uploaded to the IDR for all conditions with a discharge date in January 2013 and July 2013 (as of December 2014) (Pg.3). **Figure A3:** Modeling approach (Pg.3). **Figure A4.** Timing of calculating monthly outcomes (Pg.4). **Table A2.** Final model specification for prediction of number of admissions, readmission rate, and observation-stay rate in AMI cohort (Pg.4). **Table A3:** Specifications of the final real-time reporting models for Heart failure and pneumonia (Pg.6). **Table A4:** Results of look-back validation where we compare the rates estimated (for the months July 2016 through December 2016) in February 2017 using RTR models with the final rates later observed using data downloaded from the IDR in February 2018 for AMI, HF and Pneumonia (Pg.7). **Figure A5.** Prediction and look-back validation for heart failure admission, readmission and observation stays (July 2016–December 2016) (Pg.8). **Figure A6.** Prediction and look-back validation for pneumonia admission, readmission and observation stays (July 2016–December 2016 (Pg.9).

## Data Availability

The datasets used during the current study are available from CMS’s Integrated Data Repository, but restrictions apply to the availability of these data, which were used under contract for the current study, and so are not publicly available. The specific data analyzed for this study are included in the supplementary information files.

## Abbreviations

AMI: Acute Myocardial Infraction
HF: Heart Failure
CMS: Centers for Medicare & Medicaid Services
HRRP: Hospital Readmission Reduction Program
FFS: Fee-For-Service
ARIMA: autoregressive integrated moving average
ICD-9-CM: International Classification of Diseases, Ninth Revision, Clinical Modification
ICD-10-CM: International Classification of Diseases, Tenth Revision, Clinical Modification
IDR: Integrated Data Repository
DME: Durable medical equipment
HCPCS: Healthcare Common Procedure Coding System
AIC: Akaike information criterion
BIC: Bayesian information criterion
RMSE: Root mean square error
CI: Confidence intervals
