# Supplementary material for: Timely estimation of National Admission, readmission, and observation-stay rates in medicare patients with acute myocardial infarction, heart failure, or pneumonia using near real-time claims data
Source: BMC Health Serv Res. 2020 Aug 10;20:733. doi: 10.1186/s12913-020-05611-w (PMC7416804; doi:10.1186/s12913-020-05611-w)
Supplement: Supplementary file 1 — Additional file 1: Figure A1. Monthly cohort definition for AMI, HF or Pneumonia Readmissions and Observation Stays (Pg.2). Figure A2. Hierarchy for multiple post-discharge care events (Pg. 2). Table A1. Cumulative numbers and percentages of final action inpatient claims uploaded to the IDR for all conditions with a discharge date in January 2013 and July 2013 (as of December 2014) (Pg.3). Figure A3: Modeling approach (Pg.3). Figure A4. Timing of calculating monthly outcomes (Pg.4). Table A2. Final model specification for prediction of number of admissions, readmission rate, and observation-stay rate in AMI cohort (Pg.4). Table A3: Specifications of the final real-time reporting models for Heart failure and pneumonia (Pg.6). Table A4: Results of look-back validation where we compare the rates estimated (for the months July 2016 through December 2016) in February 2017 using RTR models with the final rates later observed using data downloaded from the IDR in February 2018 for AMI, HF and Pneumonia (Pg.7). Figure A5. Prediction and look-back validation for heart failure admission, readmission and observation stays (July 2016–December 2016) (Pg.8). Figure A6. Prediction and look-back validation for pneumonia admission, readmission and observation stays (July 2016–December 2016 (Pg.9). [file 12913_2020_5611_MOESM1_ESM.docx]

# **Appendix**

# For Manuscript by Li, et. al.: “Timely Estimation of National Admission, Readmission, and Observation-Stay Rates in Medicare Patients with Acute Myocardial Infarction, Heart Failure, or Pneumonia Using Near Real-Time Claims Data”

Below we present supplemental Figures and Tables; they are presented in order of reference in the main manuscript.

**Figure A1**. Monthly cohort definition for AMI, HF or Pneumonia Readmissions and Observation Stays (Pg.2)

**Figure A2.** Hierarchy for multiple post-discharge care events (Pg. 2)

**Table A1.** Cumulative numbers and percentages of final action inpatient claims uploaded to the IDR for all conditions with a discharge date in January 2013 and July 2013 (as of December 2014) (Pg.3)

**Figure A3:** Modeling approach (Pg.3)

**Figure A4.** Timing of calculating monthly outcomes (Pg.4)

**Table A2.** Final model specification for prediction of number of admissions, readmission rate, and observation-stay rate in AMI cohort (Pg.4)

**Table A3:** Specifications of the final real-time reporting models for Heart failure and pneumonia (Pg.6)

**Table A4:** Results of look-back validation where we compare the rates estimated (for the months July 2016 through December 2016) in February 2017 using RTR models with the final rates later observed using data downloaded from the IDR in February 2018 for AMI, HF and Pneumonia

(Pg.7)

**Figure A5.** Prediction and look-back validation for heart failure admission, readmission and observation stays (July 2016–December 2016) (Pg.8)

**Figure A6.** Prediction and look-back validation for pneumonia admission, readmission and observation stays (July 2016–December 2016 (Pg.9)

**Figure A1. Exclusion criteria for monthly cohort definitions** **for AMI, HF or Pneumonia Readmissions and Observation Stays**

**Figure A2. Hierarchy for multiple post-discharge care events**

**Table A1**. **Cumulative numbers and percentages of final action inpatient claims uploaded to the IDR for all conditions with a discharge date in January 2013 and July 2013 (as of December 2014)**

| **Follow-up Month** | **Effective Date** | **Total Number of Final Action Claims (N)** | **Cumulative Percentage of Final Action Claims (%)** |
| --- | --- | --- | --- |
| 1 | Feb 1, 2013 | 338,413 | 30.4 |
| 2 | Mar 1, 2013 | 413,691 | 37.2 |
| 3 | Apr 1, 2013 | 458,548 | 41.2 |
| 4 | May 1, 2013 | 646,708 | 58.1 |
| 5 | Jun 1, 2013 | 1,025,021 | 92.2 |
| 6 | Jul 1, 2013 | 1,051,619 | 94.6 |
| 7 | Aug 1, 2013 | 1,063,207 | 95.6 |
| 8 | Sep 1, 2013 | 1,072,936 | 96.5 |
| 9 | Oct 1, 2013 | 1,079,133 | 97.0 |
| 10 | Nov 1, 2013 | 1,086,152 | 97.7 |
| 11 | Dec 1, 2013 | 1,090,856 | 98.1 |
| 12 | Jan 1, 2014 | 1,094,132 | 98.4 |

Cumulative numbers and percentages of final action inpatient claims available at different follow-up months for patients discharged in January 2013 and July 2013. This examination is based on IDR data downloaded in December 2014.

**Figure A3: Modeling approach**

| Calendar  Month | Jan  2006 | … | May  2016 | Jun  2016 | Jul  2016 | Aug  2016 | Sep  2016 | Oct  2016 | Nov  2016 | Dec  2016 | Jan  2017 | Feb 2017 |
| --- | --- | --- | --- | --- | --- | --- | --- | --- | --- | --- | --- | --- |
| *t* | 1 | … | 125 | 126 | 127 | 128 | 129 | 130 | 131 | 132 | 133 | 134 |
| *R_m_(t)* | ***R_8_(1)*** |  | ***R_8_(125)*** | ***R_8_(126)*** | ${\hat{\boldsymbol{R}}}_{\boldsymbol{8}}\boldsymbol{(127)}$ | ${\hat{\boldsymbol{R}}}_{\boldsymbol{8}}\boldsymbol{(128)}$ | ${\hat{\boldsymbol{R}}}_{\boldsymbol{8}}\boldsymbol{(129)}$ | ${\hat{\boldsymbol{R}}}_{\boldsymbol{8}}\boldsymbol{(130)}$ | ${\hat{\boldsymbol{R}}}_{\boldsymbol{8}}\boldsymbol{(131)}$ | ${\hat{\boldsymbol{R}}}_{\boldsymbol{8}}\boldsymbol{(132)}$ |  |  |
|  | *R_7_(1)* |  | *R_7_(125)* | *R_7_(126)* | *R_7_(127)* |  |  |  |  |  |  |  |
|  | *R_6_(1)* | … | *R_6_(125)* | *R_6_(126)* | *R_6_(127)* | *R_6_(128)* |  |  |  |  |  |  |
|  | *R_5_(1)* | … | *R_5_(125)* | *R_5_(126)* | *R_5_(127)* | *R_5_(128)* | *R_5_(129)* |  |  |  |  |  |
|  | *R_4_(1)* | … | *R_4_(125)* | *R_4_(126)* | *R_4_(127)* | *R_4_(128)* | *R_4_(129)* | *R_4_(130)* |  |  |  |  |
|  | *R_3_(1)* | … | *R_3_(125)* | *R_3_(126)* | *R_3_(127)* | *R_3_(128)* | *R_3_(129)* | *R_3_(130)* | *R_3_(131)* |  |  |  |
|  | *R_2_(1)* | … | *R_2_(125)* | *R_2_(126)* | *R_2_(127)* | *R_2_(128)* | *R_2_(129)* | *R_2_(130)* | *R_2_(131)* | *R_2_(132)* |  |  |

Rows in the table are shaded to correspond to the month listed in the header row; the header row months represent the month for which that model was used to estimate the outcome. Figure depicts the prediction dates from July 2016 to December 2016 using “look-back validation” data from the IDR downloaded in February 2017.**Figure A4. Timing of calculating monthly outcomes**

**Table A2. Final model specifications for prediction of number of admissions, readmission rate, and observation-stay rate in AMI cohort**

| **Outcome** | **Value to be predicted** | **Log transformation of Outcome?** | **sARIMA terms** | **Additional Covariates** |
| --- | --- | --- | --- | --- |
| *Number of Monthly Admissions* | *D_8_(c-7)* | No | ARIMA(1,0,0) with zero mean | *D_7_(t)* |
|  | *D_8_(c-6)* | No | ARIMA(1,0,0) with non-zero mean | *D_6_(t)* |
|  | *D_8_(c-5)* | No | ARIMA(0,0,1) with zero mean | *D_5_(t), H_d_(t)* |
|  | *D_8_(c-4)* | No | ARIMA(1,0,0) with zero mean | *D_4_(t), H_d_(t)* |
|  | *D_8_(c-3)* | No | ARIMA(1,1,1) with zero mean | *D_3_(t)* |
|  | *D_8_(c-2)* | No | ARIMA(2,0,0)(1,0,0)[12] with non-zero mean | *D_2_(t), Hd(t)* |
| *Monthly 30-day Readmission Rate* | *R_8_(c-7)* | No | ARIMA(0,0,0)(2,1,0)[12] with zero mean | *R_7_(t)* |
|  | *R_8_(c-6)* | No | ARIMA(0,0,0) with zero mean | *R_6_(t)* |
|  | *R_8_(c-5)* | No | ARIMA(0,0,1) with zero mean | *R_5_(t)* |
|  | *R_8_(c-4)* | No | ARIMA(1,0,0) with non-zero mean | *R_4_(t)* |
|  | *R_8_(c-3)* | No | ARIMA(0,0,1)(0,0,1)[12] with non-zero mean | *R_3_(t)* |
|  | *R_8_(c-2)* | No | ARIMA(0,1,1)(1,0,1)[12] with zero mean | *R_2_(t)* |
| *Monthly Post-Discharge Observation-stay Rate* | *O_8_(c-7)* | No | ARIMA(0,0,0) with zero mean | *O_7_(t)* |
|  | *O_8_(c-6)* | No | ARIMA(0,0,0) with zero mean | *O_6_(t)* |
|  | *O_8_(c-5)* | No | ARIMA(0,0,0) with zero mean | *O_5_(t)* |
|  | *O_8_(c-4)* | No | ARIMA(0,0,0) with zero mean | *O_4_(t)* |
|  | *O_8_(c-3)* | No | ARIMA(0,0,0) with zero mean | *O_3_(t)* |
|  | *O_8_(c-2)* | No | ARIMA(0,0,0) with non-zero mean | *O_2_(t),* *R_2_(t),* *D_2_(t)* |

**Table A3: Specifications of the final real-time reporting models for Heart failure and pneumonia**

|  |  | **Heart Failure** | | | **Pneumonia** | | |
| --- | --- | --- | --- | --- | --- | --- | --- |
| **Outcome** | **Value to be predicted** | **Log transformation of Outcome?** | **sARIMA terms** | **Additional Covariates** | **Log transformation of Outcome?** | **sARIMA terms** | **Additional Covariates** |
| *Number of Monthly Admissions* | *D_8_(c-7)* | No | ARIMA(0,0,0) with zero mean | *D_7_(t)* | No | ARIMA(0,0,0) with zero mean | *D_7_(t)* |
|  | *D_8_(c-6)* | No | ARIMA(1,0,0) with zero mean | *D_6_(t)* | No | ARIMA(1,0,0) with zero mean | *D_6_(t)* |
|  | *D_8_(c-5)* | No | ARIMA(0,0,1) with zero mean | *D_5_(t)* | No | ARIMA(0,0,1) with zero mean | *D_5_(t)* |
|  | *D_8_(c-4)* | No | ARIMA(2,0,0) with zero mean | *D_4_(t)* | No | ARIMA(1,0,1) with zero mean | *D_4_(t), H_d_(t)* |
|  | *D_8_(c-3)* | No | ARIMA(1,0,0)(1,0,0)[12] with zero mean | *D_3_(t), H_d_(t)* | No | ARIMA(2,0,0) with zero mean | *D_3_(t), H_d_(t)* |
|  | *D_8_(c-2)* | No | ARIMA(0,0,0)(1,0,0)[12] with non-zero mean | *D_2_(t), H_d_(t)* | No | ARIMA(1,0,0) with zero mean | *D_2_(t)* |
| *Monthly 30-day Readmission Rate* | *R_8_(c-7)* | No | ARIMA(1,0,0) with zero mean | *R_7_(t)* | No | ARIMA(0,0,0) with zero mean | *R_7_(t), H_d_(t)* |
|  | *R_8_(c-6)* | No | ARIMA(1,0,0) with zero mean | *R_6_(t),*  *H_r_(t)* | No | ARIMA(0,0,0)(0,0,1)[12] with zero mean | *R_6_(t),*  *H_r_(t)* |
|  | *R_8_(c-5)* | No | ARIMA(1,0,0) with zero mean | *R_5_(t)* | No | ARIMA(0,0,1) with zero mean | *R_5_(t)* |
|  | *R_8_(c-4)* | No | ARIMA(1,0,0)(1,0,0)[12] with non-zero mean | *R_4_(t)* | No | ARIMA(0,0,1) with zero mean | *R_4_(t), H_r_(t)* |
|  | *R_8_(c-3)* | No | ARIMA(1,0,0)(1,0,0)[12] with non-zero mean | *R_3_(t)* | No | ARIMA(1,0,0)(0,0,1)[12] with zero mean | *R_3_(t)* |
|  | *R_8_(c-2)* | No | ARIMA(0,1,1)(1,0,1)[12] with zero mean | *R_2_(t)* | No | ARIMA(2,0,0)(2,0,0)[12] with non-zero mean | *R_2_(t)* |
| *Monthly Post-Discharge Observation-Stay Rate* | *O_8_(c-7)* | No | ARIMA(1,0,0) with zero mean | *O_7_(t), D_7_(t)* | No | ARIMA(0,0,0)(0,1,1)[12] with zero mean | *O_7_(t), R_7_(t)* |
|  | *O_8_(c-6)* | No | ARIMA(1,0,0) with zero mean | *O_6_(t), D_6_(t)* | No | ARIMA(0,0,0) with zero mean | *O_6_(t), D_6_(t)* |
|  | *O_8_(c-5)* | No | ARIMA(1,0,0) with zero mean | *O_5_(t)* | No | ARIMA(0,0,0) with zero mean | *O_5_(t), R_5_(t)* |
|  | *O_8_(c-4)* | No | ARIMA(1,0,0)(1,0,0) with non-zero mean | *O_4_(t)* | No | ARIMA(0,0,0) with zero mean | *O_4_(t)* |
|  | *O_8_(c-3)* | No | ARIMA(1,0,1)(1,0,0) with non-zero mean | *O_3_(t)* | Yes | ARIMA(0,1,1)(0,0,1)[12] with zero mean | *O_3_(t)* |
|  | *O_8_(c-2)* | No | ARIMA(0,1,1)(1,0,1) with zero mean | *O_2_(t)* | Yes | ARIMA(2,1,0)(2,0,0)[12] with zero mean | *O_2_(t)* |

ARIMA = autoregressive integrated moving average

**Table A4: Results of look-back validation**

This table presents results comparing the rates estimated (for months July 2016 through December 2016) in February 2017 using the final prediction models with the final rates later observed using data downloaded from the IDR in February 2018 for AMI, HF and Pneumonia.

| **Discharge Month** | **Number of admissions** | | **Readmission rate** | | **Observation-Stay Rate** | |
| --- | --- | --- | --- | --- | --- | --- |
|  | Original Estimated (95% CI) | **2018 IDR Download** (True) | Original Estimated (95% CI) | **2018 IDR Download** (True) | Original Estimated (95% CI) | **2018 IDR Download** (True) |
| **Acute Myocardial Infarction** | | | | | | |
| 7/1/2016 | 14595 (14573-14616) | 14597 | 16.18 (16.14-16.22) | 16.14 | 3.11 (3.10-3.13) | 3.13 |
| 8/1/2016 | 15200 (15149-15250) | 15202 | 15.48 (15.42-15.54) | 15.51 | 3.19 (3.16-3.21) | 3.18 |
| 9/1/2016 | 14925 (14842-15007) | 14913 | 15.98 (15.90-16.06) | 16.00 | 3.42 (3.39-3.46) | 3.44 |
| 10/1/2016 | 15528 (15419-15637) | 15552 | 15.90 (15.76-16.04) | 15.86 | 3.15 (3.10-3.21) | 3.13 |
| 11/1/2016 | 15890 (15718-16062) | 15923 | 16.20 (15.92-16.48) | 16.01 | 3.36 (3.25-3.48) | 3.24 |
| 12/1/2016 | 17050 (16653-17446) | 17028 | 16.04 (15.12-16.96) | 16.62 | 2.83 (2.63-3.04) | 2.92 |
| **Heart Failure** | | | | | | |
| 7/1/2016 | 33417 (33374-33461) | 33407 | 22.37 (22.34-22.40) | 22.40 | 2.25 (2.23-2.26) | 2.24 |
| 8/1/2016 | 33226 (33107-33345) | 33230 | 22.71 (22.66-22.77) | 22.74 | 2.27 (2.25-2.29) | 2.27 |
| 9/1/2016 | 33114 (32918-33310) | 33117 | 22.39 (22.30-22.48) | 22.45 | 2.40 (2.37-2.43) | 2.38 |
| 10/1/2016 | 36209 (35947-36471) | 36237 | 22.22 (22.08-22.35) | 22.29 | 2.26 (2.22-2.31) | 2.24 |
| 11/1/2016 | 37668 (37244-38093) | 37676 | 22.36 (22.05-22.67) | 22.38 | 2.18 (2.10-2.26) | 2.19 |
| 12/1/2016 | 43029 (42043-44015) | 42845 | 22.31 (21.59-23.03) | 22.62 | 2.25 (2.00-2.51) | 2.12 |
| **Pneumonia** | | | | | | |
| 7/1/2016 | 35906 (35781-36031) | 35884 | 18.02 (17.99-18.04) | 18.06 | 1.72 (1.71-1.73) | 1.71 |
| 8/1/2016 | 35086 (34655-35517) | 35060 | 17.85 (17.80-17.90) | 17.93 | 1.68 (1.66-1.69) | 1.66 |
| 9/1/2016 | 36654 (35617-37691) | 36596 | 17.99 (17.91-18.08) | 18.04 | 1.90 (1.88-1.92) | 1.88 |
| 10/1/2016 | 37386 (36060-38713) | 37343 | 17.27 (17.13-17.41) | 17.23 | 1.70 (1.66-1.73) | 1.70 |
| 11/1/2016 | 37047 (35594-38500) | 37003 | 17.49 (17.19-17.79) | 17.45 | 1.80 (1.74-1.86) | 1.76 |
| 12/1/2016 | 44716 (42769-46663) | 44589 | 17.79 (16.82-18.76) | 17.61 | 1.60 (1.43-1.77) | 1.66 |

CMS = Centers for Medicare & Medicaid Services; IDR = Integrated Data Repository.

**Figure A5. Prediction and look-back validation for heart failure admission, readmission and observation stays (July 2016–December 2016) – These correspond to Figures 2A-F (AMI) in the Main Manuscript**

**Figure A6. Prediction and look-back validation for pneumonia admission, readmission and observation stays (July 2016–December 2016) – These correspond to Figures 2A-F (AMI) in the Main Manuscript**
